# IL‐12 minicircle delivery via extracellular vesicles as immunotherapy for bladder cancer

**DOI:** 10.1111/cpr.13739

**Published:** 2024-08-28

**Authors:** Zhiyuan Wu, Wei Li, Melissa Tan, Faith Yuan Xin How, Haripriya Sadhasivan, Ratha Mahendran, Qinghui Wu, Edmund Chiong, Minh T. N. Le

**Affiliations:** ^1^ Department of Pharmacology and Institute for Digital Medicine, Yong Loo Lin School of Medicine National University of Singapore Singapore Singapore; ^2^ Department of Surgery, Yong Loo Lin School of Medicine National University of Singapore Singapore Singapore; ^3^ Shanghai University of Medicine & Health Sciences Shanghai China; ^4^ Jiading District Central Hospital Affiliated to Shanghai University of Medicine & Health Sciences Shanghai China; ^5^ Carmine Therapeutics Singapore Singapore; ^6^ Department of Urology National University Hospital Singapore Singapore; ^7^ Institute of Molecular and Cell Therapy, A*STAR Singapore Singapore

## Abstract

Interleukin‐12 (IL‐12) holds significant potential in cancer therapy; however, its clinical applicability is hindered by dose‐limiting toxicity. Delivery of the *IL‐12* gene directly to tumours for constitutive IL‐12 expression is a possible strategy to enhance its effectiveness while minimizing systemic toxicity. In this study, we investigate the potential of red blood cell‐derived extracellular vesicles (RBCEVs) as a carrier for *Il‐12* plasmid delivery. We demonstrate that RBCEVs can be loaded with minicircle plasmid encoding IL‐12 and delivered to MB49 bladder cancer cells for IL‐12 expression. The expression of transgenes from minicircles was significantly higher than from the parental plasmids. RBCEV‐mediated IL‐12 expression stimulated immune responses in mouse splenocytes. Intratumoral delivery of *Il‐12* plasmid‐loaded RBCEVs suppressed bladder cancer tumour growth, stimulated immune responses and promoted immune cell infiltration. In conclusion, our study demonstrates the promising potential of RBCEVs as an effective, safe and redosable nucleic acid drug delivery platform for IL‐12.

## Graphical abstract

RBCEVs can be loaded with minicircle plasmid encoding IL‐12 and delivered to MB49 bladder cancer cells for functional IL‐12 expression. RBCEV‐mediated intratumoral delivery of IL‐12 plasmid suppressed bladder cancer tumour growth, stimulated immune responses and promoted immune cell infiltration.

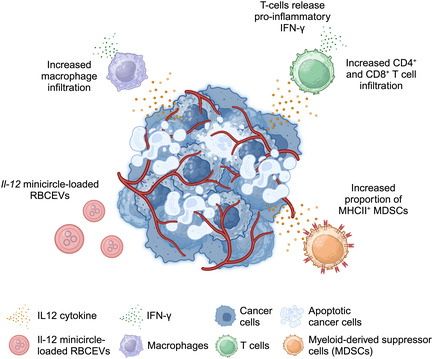

## 1 INTRODUCTION

The immune system plays a crucial role in mounting effective antitumor responses. However, cancer has demonstrated its ability to evade these defences through various mechanisms. Immunotherapy has been a revolutionary tool against cancer, aiming to enhance host immune system responses to fight the disease.^1^ Interleukin 12 (IL‐12) is a potent pro‐inflammatory cytokine that has been employed in immunotherapy against several types of cancer.^2^, ^3^, ^4^ Among its multifaceted functions, IL‐12 is mainly responsible for activating T helper 1 (Th1) cell‐mediated inflammatory responses and the secretion of interferon‐γ (IFNγ) by Th1 cells.^2^, ^3^, ^4^ Treatment of tumours with IL‐12 has been shown to activate pro‐inflammatory responses, increase immune cell infiltration into tumours and counteract the immunosuppressive tumour microenvironment.^2^, ^3^, ^4^

Despite demonstrating substantial antitumor efficacy in numerous preclinical cancer models, the dose‐limiting toxicity of IL‐12 hampers its clinical applicability. In clinical trials, therapeutic doses of IL‐12 have resulted in considerable toxicity and systemic delivery of recombinant IL‐12 has led to severe adverse reactions.^5^, ^6^, ^7^ Additionally, the limited effectiveness of IL‐12 may stem partly from the insufficient delivery to tumour tissues.^3^, ^8^ This underscores the need for novel approaches to enhance delivery and sustain the expression of IL‐12 within the tumour microenvironment, while reducing toxicity in surrounding healthy tissues. Local delivery of IL‐12 would help enhance tumour targeting, reduce systemic toxicity and would require lower doses compared to systemic administration.^2^, ^3^

Gene therapy offers a promising avenue for the delivery of IL‐12 for prolonged expression. Commonly used gene delivery vehicles encompass both viral and non‐viral vectors.^9^ However, viral vectors are substantially limited by their immunogenicity, often restricting these therapeutics to single‐dose administration due to potential neutralization by the host immune system in subsequent doses.^9^, ^10^ Viral vectors have undergone many developments to optimize their use as delivery vectors, yet safety concerns persist with reports of complications in clinical trials.^9^, ^10^ Non‐viral delivery vectors, notably lipid nanoparticles (LNPs) and cationic polymer‐based vehicles, face their own sets of challenges including immunogenicity and toxicity.^9^, ^11^

We have recently shown that red blood cell‐derived extracellular vesicles (RBCEVs) are a promising vector for gene therapy. Obtained from human red blood cells (RBCs), RBCEVs are devoid of genetic materials and pose no risk of horizontal gene transfer.^12^ As they are produced naturally in human bodies, they are biocompatible, non‐immunogenic and readily available from healthy donors.^12^ Additionally, RBCEVs have demonstrated robust delivery of mRNA and antisense oligonucleotides to various cell types.^13^, ^14^, ^15^ Given the ability of IL‐12 to modulate the tumour microenvironment, we aim to deliver IL‐12‐encoding plasmids using RBCEVs to bladder cancer cells.

Bladder cancer is one of the most prevalent malignancies in the urinary system with an estimated 83,190 new cases and 16,840 deaths in the United States in 2024.^16^ It encompasses a spectrum of conditions, ranging from non‐muscle‐invasive bladder cancer (NMIBC) that is characterized by frequent recurrence, to muscle‐invasive and advanced stage disease.^17^, ^18^ The gold standard treatment for NMBIC is Bacillus Calmette Guerin (BCG); however, 30%–50% of patients do not respond to this therapy.^17^, ^18^ Thus, there is a need for alternative treatment options.

In this study, we aimed to deliver IL‐12‐encoding plasmids using RBCEVs for bladder cancer treatment. Indeed, plasmid delivery is more challenging than small RNA/DNA delivery studies, as we illustrated in our previous reports, due to the larger size of plasmid DNA. Moreover, large DNA like plasmids often trigger innate immune responses, such as the cGAS and STING pathways, that sequester transgene expression. Therefore, we sought to use minicircle plasmids in which we remove bacterial elements to reduce the size of the plasmid and to minimize immunogenicity. We demonstrated that *Il‐12* plasmids can be loaded on RBCEVs and delivered to MB49 bladder cancer cells for functional IL‐12 secretion. Expression of transgenes from minicircles was significantly higher than from the parental plasmids. Furthermore, intratumoral delivery of *Il‐12* minicircle‐loaded RBCEVs to mice bearing MB49 bladder cancer in the flank region suppressed tumour growth, stimulated immune responses and promoted immune cell infiltration.

## 2 MATERIALS AND METHODS

### 2.1 Cell culture

Human HEK293T and mouse MB49 cells were obtained from American Type Culture Collection (ATCC, USA). All cells were cultured in Dulbecco's Modified Eagle Media (Thermo Fisher Scientific, USA) supplemented with 10% foetal bovine serum (FBS), 1% penicillin/streptomycin (Thermo Fisher Scientific) and 5 μg/mL Plasmocin prophylactic (InvivoGen, USA). Cells were maintained in an incubator at 37°C with 5% CO_2_.

### 2.2 Purification of RBCEVs

Type O negative blood from healthy donors with informed consent was obtained by ESCO Aster (Singapore) from blood banks in the USA via iDNA (Singapore). Red blood cells (RBCs) were separated from plasma by centrifugation at 1000 ***g*** for 8 min at 4°C and washed with PBS thrice. White blood cells were removed by passing through leukodepletion filters (Nigale, China). Flowthrough containing RBCs was collected in Nigale buffer with addition of 0.1 mg/mL calcium chloride and 10 μM calcium ionophore (Sigma‐Aldrich, USA) then incubated overnight at 37°C and 5% CO_2_ for induction of vesicles. RBCEVs were purified as previously described.^19^ Purified RBCEVs were resuspended in PBS and stored with 4% trehalose at −80°C.

### 2.3 Cloning and minicircle production

A mouse IL‐12 encoding plasmid was obtained from Addgene (USA, plasmid #108665). The GFP encoding plasmid was obtained from AAV GFP (Addgene, plasmid #49055). *IL‐12* and *GFP* genes were cloned into the pMC.EF1α‐MCS‐SV40polyA Parental Minicircle Cloning Vector (System Biosciences, USA) using the multiple cloning site. Parental plasmids were used for minicircle production using the MC‐Easy™ Minicircle DNA Production Kit with ZYCY10P3S2T Minicircle Production Competent Cells (System Biosciences) in accordance with the manufacturer's protocols. Minicircles were extracted using QIAGEN Plasmid Midi Kit following the manufacturer's protocol. The purity of minicircle plasmids was verified by restriction enzyme digestion and gel electrophoresis.

### 2.4 Nanoparticle tracking analysis

The size distribution and concentration of RBCEVs were quantified utilizing a ZetaView® Particle Tracking Analysis instrument (Particle Metrix, Germany). The quantity of RBCEVs was reported based on haemoglobin content throughout this study as haemoglobin is the major component of RBCEVs. Haemoglobin in RBCEVs was quantified using a NanoDrop™ 2000 spectrophotometer (Thermo Fisher Scientific).

### 2.5 Plasmid loading into RBCEVs

A total of 20 μg of RBCEVs was loaded with 1 μg of plasmid DNA using the REG1 reagent (Carmine Therapeutics, USA) in accordance with the manufacturer's protocol. Free DNA and REG1 were washed away with PBS in three rounds of centrifugation at 18,500 ***g*** for 45 min.

### 2.6 Quantification of DNA loaded in RBCEVs

After DNA loading, RBCEVs were lysed with 1% Triton‐X for 5 min at room temperature and incubated with heparin sulphate (20 mg/mL final concentration) for 1 h at 37°C. The mixture was loaded onto 1% agarose gel with GelRed® Nucleic Acid Gel Stain (Sigma‐Aldrich), separated at 100 V for 30 min and visualized with ChemiDoc Imaging System (Bio‐Rad, USA). The band fluorescence intensity was quantified using ImageJ software.

### 2.7 In vitro treatment of cells with RBCEVs

A total of 50,000 HEK293T cells or MB49 cells were seeded in a 24‐well plate and incubated with 20 μg of RBCEVs or DNA‐loaded RBCEVs for different durations in an incubator at 37°C with 5% CO_2_. Cells were harvested for flow cytometry, RT‐qPCR or ELISA analysis.

### 2.8 Flow cytometry analysis

Cells were washed twice and suspended in FACS buffer (2% FBS in PBS). SYTOX™ Blue Dead Cell Stain (Invitrogen, USA) was added to cells prior to analysis with a CytoFLEX LX Flow Cytometer (Beckman Coulter, USA). Data analysis was conducted using FlowJo V10.

#### 2.8.1 Splenocyte and tumour immune cell analysis

Mouse spleen and tumours were harvested and dissociated in DMEM supplemented with 10% FBS and 5 mg/mL collagenase IV (Thermo Fisher Scientific) using the gentleMACS™ Dissociator (Miltenyi Biotec, Germany). The cell suspension was filtered using a 70 μm strainer and blocked with anti‐mouse TruStain FcX™ (anti‐mouse CD16/32) antibody (Biolegend). Different populations of immune cells were stained with the following antibodies: anti‐mouse CD3ε‐PE (Biolegend, Cat# 100307), anti‐mouse CD4‐APC/Fire™ 750 (Biolegend, Cat# 100460), anti‐mouse CD8a‐FITC (Biolegend, Cat# 100706), anti‐mouse CD49b‐APC (Biolegend, Cat# 103516), anti‐mouse/human CD11b‐FITC (Biolegend, Cat# 101206), anti‐mouse F4/80‐APC (Biolegend, Cat# 123116), anti‐mouse CD80‐PE (Biolegend, Cat# 104707), anti‐mouse Gr‐1‐APC (Biolegend, Cat# 108411) and anti‐mouse I‐A/I‐E‐PE (Biolegend, Cat# 107607). Compensation was performed using BD™ CompBeads Anti‐Rat and Anti‐Hamster Ig κ/Negative Control Compensation Particles Set (BD Biosciences, USA, Cat# 552845).

### 2.9 RNA extraction and qRT‐PCR

Total RNA was extracted from cells using TRIzol® (Thermo Fisher Scientific) following the manufacturer's protocol. cDNA was synthesized from RNA using a High‐Capacity cDNA Reverse Transcription Kit (Thermo Fisher Scientific) in accordance with the manufacturer's protocols. mRNA levels were quantified using SsoAdvanced™ Universal SYBR® Green Supermix (Bio‐Rad), and expression levels were normalized to mouse *Gapdh*. Primer sequences are provided in Table S1. qPCR reactions were conducted using a QuantStudio™ 6 Real‐Time PCR system (Life Technologies, USA).

### 2.10 ELISA

Cell culture supernatant was collected for analysis. Cytokines were measured using the ELISA MAX™ Deluxe Set Mouse IL‐12 (p70) kit and ELISA MAX™ Deluxe Set Mouse IFN‐γ kit (Biolegend) according to the manufacturer's protocol. Absorbance was measured using Spark 10M multimode plate reader (Tecan, Switzerland).

### 2.11 Tumour model and intratumoral administration of RBCEVs

All mouse experiments were performed according to experimental protocols approved by the Institutional Animal Care and Use Committee of the National University of Singapore. C57BL/6 mice, aged 6‐8 weeks, were subcutaneously injected with 1 × 10^6^ MB49 cells in the flank pad. When tumour diameter reached ~4 mm, mice were randomly grouped and treated with 400 μg *Il‐12* plasmid‐loaded RBCEVs by intratumoral injection. Tumours were treated every two days for a total of six doses. Mice were euthanized 18 days after the first dose, when control tumours reached ∼15 mm in diameter.

### 2.12 Statistical analysis

Student's one‐tailed *t*‐test and one‐way ANOVA for comparison between multiple groups were performed using GraphPad Prism 10. A *p*‐value < 0.05 was considered significant. In graphs, data are presented as mean and error bars indicating standard error of the mean (SEM).

## 3 RESULTS

### 3.1 RBCEVs can be loaded with minicircle for delivery and transgene expression in cells

**FIGURE 1:**
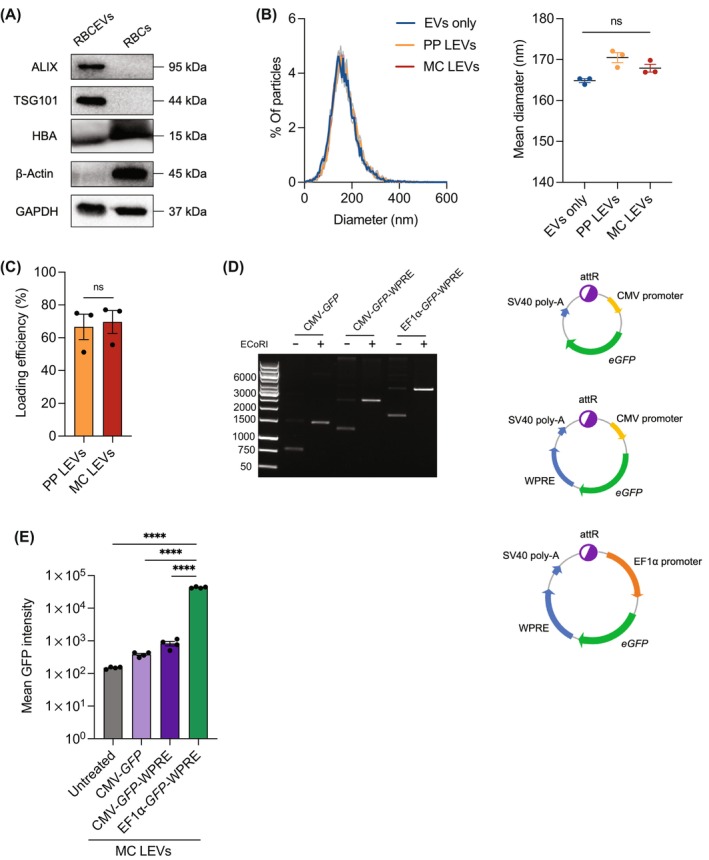
RBCEVs can be loaded with minicircle for delivery and transgene expression in cell lines. (A) Western blot analysis of ALIX, TSG101, Haemoglobin A (HBA), β‐Actin and GAPDH in the lysates of RBCs and RBCEVs. (B) Size distribution of unloaded RBCEVs (EVs only), parental plasmid (PP LEVs) and minicircle DNA‐loaded RBCEVs (MC LEVs), determined using the ZetaView® nanoparticle tracking analyser (*n* = 3). (C) Loading efficiency of parental plasmid (PP) or minicircle (MC) in RBCEVs, determined using gel electrophoresis (*n* = 3). (D) Plasmid maps and agarose gel electrophoresis images of minicircle constructs CMV‐eGFP, CMV‐eGFP‐WPRE or EF1α‐eGFP‐WPRE. (E) Flow cytometry analysis of GFP in HEK293T cells treated with RBCEVs loaded with CMV‐*eGFP*, CMV‐*eGFP*‐WPRE or EF1α‐*eGFP*‐WPRE minicircle for 48 h (*n* = 3). All bar graphs represent mean ± SEM. ns, not significant, ****p < 0.0001 determined by Student's one‐tailed *t*‐test (C) and one‐way ANOVA (B, E).

RBCEVs were purified as described in our previous study.^19^ Purified RBCEVs were shown to be enriched in EV markers ALIX and TSG101, and RBC protein haemoglobin A (HBA) (Figure 1A). Cytoskeleton protein β‐actin was absent in RBCEVs, indicating an efficient purification method that eliminated cellular contaminants (Figure 1A). For delivery, purified RBCEVs were loaded with plasmids using the loading reagent REG1 (Carmine Therapeutics, USA). Unloaded, parental plasmid‐loaded and minicircle‐loaded RBCEVs were homogenous in size, with an average diameter ranging from 160 to 170 nm as measured by nanoparticle tracking analysis (Figure 1B). The plasmid loading did not significantly change the size of RBCEVs. To estimate the efficiency of REG1‐mediated loading, plasmid‐loaded RBCEVs were treated with detergents for lysis and subsequently separated using gel electrophoresis. The band intensity indicated that approximately 70% of the DNA was successfully loaded into RBCEVs by REG1. There was no significant difference in the loading efficiency of parental plasmid and minicircle (Figure 1C).

To enhance transgene expression, we incorporated different promoters, elongation factor‐1α (EF1α) or Cytomegalovirus (CMV), and regulatory elements, woodchuck hepatitis virus posttranscriptional regulatory element (WPRE) and simian virus 40 enhancer (SV40), into the enhanced GFP (eGFP) minicircle construct. Gel electrophoresis revealed a single major band following linearization of minicircle DNA, confirming the high purity of minicircles free from parental plasmid contamination (Figure 1D). Minicircles were dominantly supercoiled and smaller than the parental plasmid (Figure 1D). RBCEVs were loaded with three different minicircles, CMV‐*eGFP*, CMV‐*eGFP*‐WPRE or EF1α‐*eGFP*‐WPRE and delivered to HEK293T cells. Flow cytometry analysis revealed that RBCEV‐mediated delivery of all three minicircles led to GFP expression and the EF1α‐*eGFP*‐WPRE construct resulted in the highest GFP expression (Figure 1E). This indicates that EF1α promoter and WPRE could enhance transgene expression and hence were included in the design for the *Il‐12* minicircle.

### 3.2 RBCEVs can deliver *Il‐12* minicircle for expression in MB49 bladder cancer cells

**FIGURE 2:**
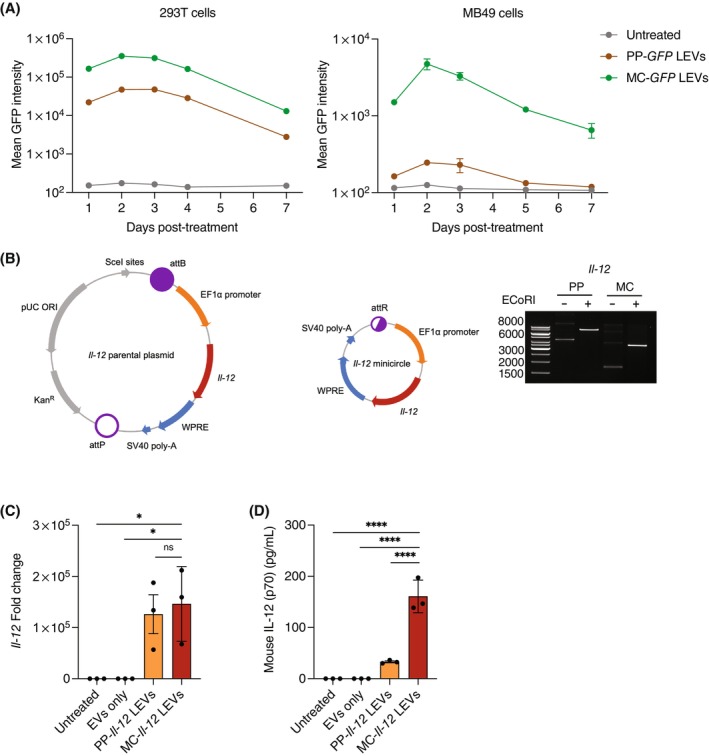
RBCEVs can deliver *Il‐12* minicircle for expression in MB49 bladder cancer cells. (A) Flow cytometry analysis of HEK293T and MB49 cells treated with RBCEVs loaded with EF1α‐*eGFP*‐WPRE parental plasmid (PP‐*GFP* LEVs) or minicircle (MC‐*GFP* LEVs) (*n* = 3). (B) Plasmid maps and agarose gel electrophoresis of *Il‐12* parental plasmid (PP) or *Il‐12* minicircle (MC). (C) qPCR analysis of *Il‐12* fold change relative to untreated, normalized to *Gapdh* in MB49 cells treated with RBCEVs loaded with *Il‐12* parental plasmid (PP‐*Il‐12* LEVs) or *Il‐12* minicircle (MC‐*Il‐12* LEVs) (*n* = 3). (D) ELISA quantification of mouse IL‐12p70 protein in culture supernatant of MB49 cells treated with RBCEVs loaded with *Il‐12* parental plasmid (PP‐*Il‐12* LEVs) or *Il‐12* minicircle (MC‐*Il‐12* LEVs) (*n* = 3). All bar graphs represent mean ± SEM. ns, not significant; *p < 0.05, ****p < 0.0001 determined by one‐way ANOVA.

To compare transgene expression of parental plasmid and minicircle, RBCEVs were loaded with *eGFP* parental plasmid or *eGFP* minicircle and delivered to HEK293T and MB49 murine bladder cancer cells. Flow cytometry analysis revealed that HEK293T and MB49 cells could express GFP after RBCEV‐mediated delivery of both parental plasmid and minicircle (Figure 2A). Notably, the delivery of *eGFP* minicircle resulted in significantly higher GFP expression compared to the parental plasmid across all timepoints (Figure 2A).

Murine *Il‐12* was cloned into the minicircle construct to generate EF1α‐*Il‐12*‐WPRE (Figure 2B). MB49 cells were transfected with RBCEVs loaded with *Il‐12* parental plasmid or *Il‐12* minicircle. qPCR analysis demonstrated that treatment with both *Il‐12* constructs led to a significant increase in *Il‐12* expression compared to controls (Figure 2C). There was no significant difference in mRNA expression between parental plasmid or minicircle (Figure 2C). In contrast, ELISA analysis revealed that the *Il‐12* minicircle construct led to 160.9 pg/mL of IL‐12 protein, approximately 5‐fold higher compared to the *Il‐12* parental plasmid (33.0 pg/mL) (Figure 2D).

**FIGURE 3:**
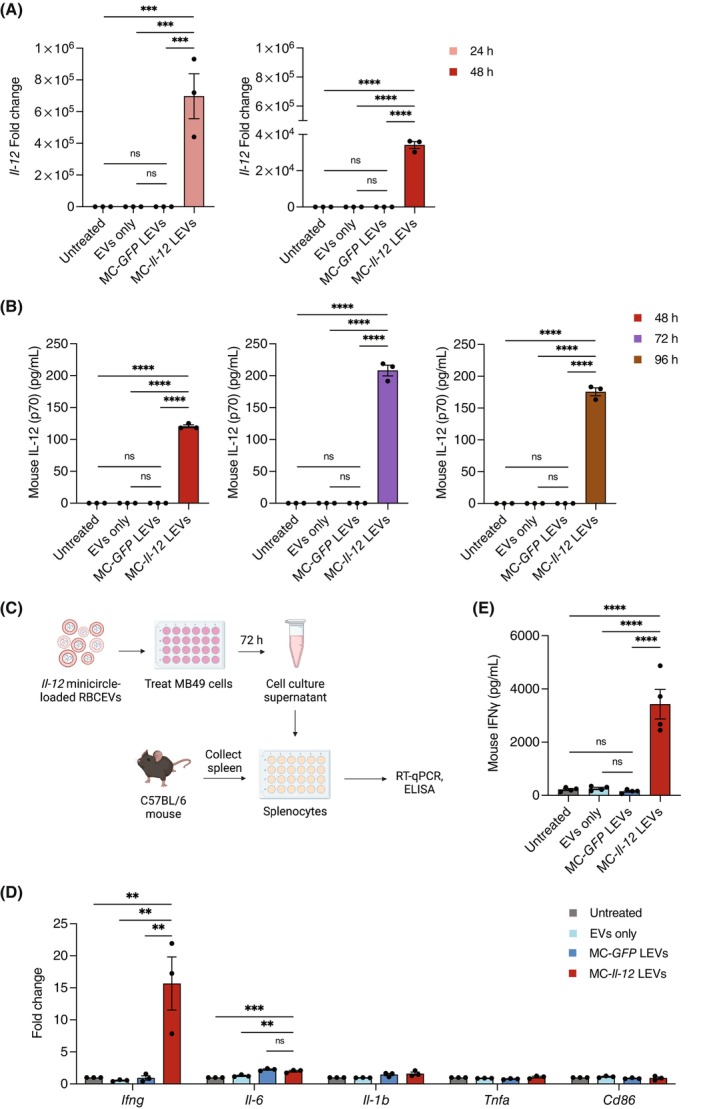
Delivery of *Il‐12* minicircle‐loaded RBCEVs can stimulate immune responses in mouse splenocytes. (A) qPCR analysis of *Il‐12* fold change relative to untreated control, normalized to *Gapdh* in MB49 cells 24 and 48 h post‐treatment (*n* = 3). (B) ELISA quantification of mouse IL‐12p70 protein in MB49 cell culture supernatant 48, 72 and 96 h post‐treatment (*n* = 3). (C) Schematic depiction of the procedure for stimulating C57BL/6 mouse splenocytes with IL‐12 secreted from MB49 cells treated with *Il‐12* minicircle‐loaded RBCEVs. (D) qPCR analysis of *Ifng*, *Il‐6*, *Il‐1b*, *Tnfa* and *Cd86* fold change relative to untreated, normalized to *Gapdh* in mouse splenocytes after treatment shown in (C) (*n* = 3). (E) ELISA quantification of mouse IFNγ protein secreted by mouse splenocytes after treatment shown in (C) (*n* = 3). All bar graphs represent mean ± SEM. ns, not significant; **p < 0.01, ***p < 0.001, and ****p < 0.0001 determined by one‐way ANOVA.

To investigate gene expression over time, MB49 cells were treated with *Il‐12* minicircle‐loaded RBCEVs for different durations. Expression of *Il‐12* mRNA was highest 24 h post‐treatment, while protein secretion peaked at 72 h post‐treatment (Figure 3A,B). Therefore, we demonstrate that RBCEVs can deliver *Il‐12* minicircle for expression and secretion of IL‐12 in MB49 cells.

### 3.3 RBCEVs can deliver *Il‐12* minicircle for expression in MB49 bladder cancer cells and stimulate immune responses in mouse splenocytes

To evaluate the function of IL‐12 delivered by *Il‐12* minicircle‐loaded RBCEVs, we used IL‐12 secreted from treated MB49 cells to stimulate mouse splenocytes. MB49 cells were treated with *Il‐12* minicircle‐loaded RBCEVs and after 72 h, the cell culture supernatant containing secreted IL‐12 was added to mouse splenocyte culture (Figure 3C). The secreted IL‐12 led to significant up‐regulation of *Ifng* mRNA and IFNγ protein secretion, as well as an increase in pro‐inflammatory cytokine *Il‐6* compared to controls in mouse splenocytes (Figure 3D,E). Overall, RBCEV‐mediated delivery of *Il‐12* minicircle led to successful IL‐12 secretion in MB49 cells and stimulated expression of downstream pro‐inflammatory cytokines in mouse splenocytes.

### 3.4 Intratumoral delivery of *Il‐12* minicircle‐loaded RBCEVs suppresses bladder cancer tumour growth

**FIGURE 4:**
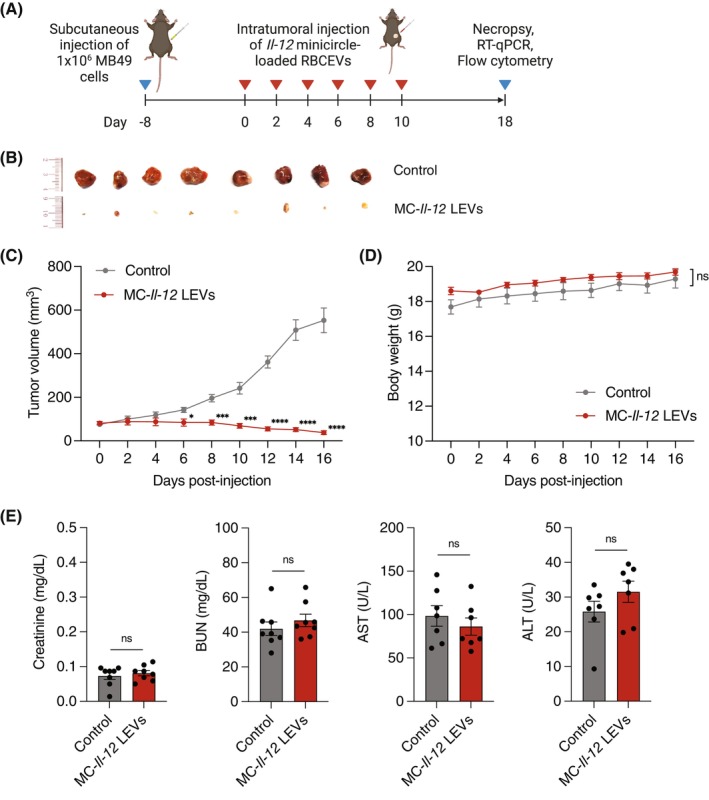
Intratumoral delivery of *Il‐12* minicircle‐loaded RBCEVs suppresses bladder cancer tumour growth. (A) Schematic showing the treatment of mouse MB49 bladder cancer model in C57BL/6 mice and intratumoral delivery of *Il‐12* minicircle‐loaded RBCEVs. (B) Tumour images at the treatment endpoint. (C) Tumour volume of mice treated with saline (vehicle control) or *Il‐12* minicircle‐loaded RBCEVs (*n* = 8). (D) Body weight of mice treated with saline (vehicle control) or *Il‐12* minicircle‐loaded RBCEVs (MC‐*Il‐12* LEVs) (*n* = 8). (E) Kidney and liver toxicity indicated by the concentration of creatinine, blood urea nitrogen (BUN), alanine aminotransferase (ALT) and aspartate aminotransferase (AST) in mice at the endpoint of treatment with saline (vehicle control) or *Il‐12* minicircle‐loaded RBCEVs (MC‐*Il‐12* LEVs) (*n* = 8). All bar graphs represent mean ± SEM. ns, not significant; *p < 0.05, ***p < 0.001, and ****p < 0.0001 determined by Student's one‐tailed *t*‐test.

Having demonstrated that *Il‐12* minicircle delivery by RBCEVs enables MB49 cancer cells to secrete biologically active cytokine, we established a subcutaneous tumour model of MB49 bladder cancer to validate antitumor efficacy in vivo (Figure 4A). MB49 cells were implanted into the flanks of C57BL/6 mice. Palpable MB49 tumours were treated with 400 μg of *Il‐12* minicircle‐loaded RBCEVs every 2 days for a total of six doses. The mice were euthanized and tumours were harvested 18 days after the first dose, when the untreated tumours reached a diameter of approximately 15 mm. We observed a significant reduction in MB49 tumour growth following treatment with *Il‐12* minicircle‐loaded RBCEVs compared to the vehicle control (Figure 4B,C). There was no significant difference in body weight between the control and *Il‐12* minicircle‐loaded RBCEVs treated groups, suggesting that the treatment did not lead to any systemic toxicity in mice (Figure 4D). To further evaluate the potential toxicity of *Il‐12* minicircle delivery by RBCEVs, mouse serum was collected at the experiment endpoint for kidney and liver function tests. There were no differences in kidney and liver toxicity markers, creatinine, urea, alanine aminotransferase (ALT) and aspartate aminotransferase (AST), between the control and *Il‐12* minicircle‐loaded RBCEVs treated mice (Figure 4E). In contrast to numerous DNA gene therapies using AAVs and LNPs, delivery of minicircle DNA by RBCEVs did not lead to observable toxicity.

### 3.5 RBCEV‐mediated intratumoral delivery of *Il‐12* minicircle stimulates immune responses and facilitates immune cell infiltration

**FIGURE 5:**
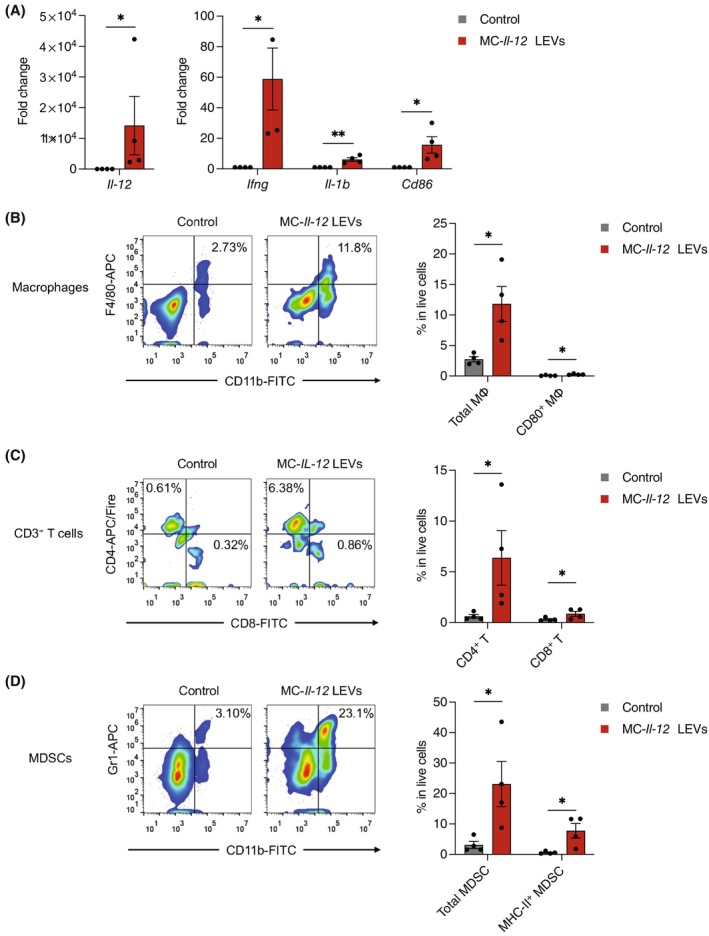
Delivery of *Il‐12* minicircle‐loaded RBCEVs stimulates immune responses and immune cell infiltration. (A) qPCR analysis of *Il‐12*, *Ifng*, *Il‐1b* and *Cd86* fold change relative to control, normalized to *Gapdh* in MB49 tumours after treatment with saline (vehicle control) or *Il‐12* minicircle‐loaded RBCEVs (MC‐*Il‐12* LEVs) (*n* = 4). (B‐D) Flow cytometry analysis of macrophages (B), T cells (C) and myeloid‐derived suppressor cells (MDSCs) (D) in MB49 tumours of mice treated with saline (vehicle control) or *Il‐12* minicircle‐loaded RBCEVs (MC‐*Il‐12* LEVs) (*n* = 4 mice). All bar graphs represent mean ± SEM. *p < 0.05, ** *p* < 0.01 determined by Student's one‐tailed *t*‐test.

**FIGURE 6:**
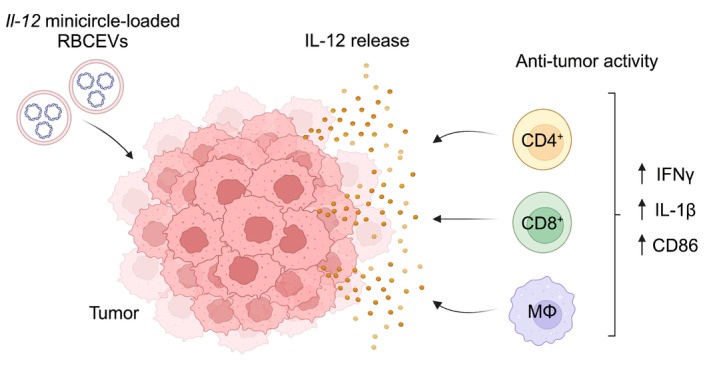
Schematic of the delivery of *Il‐12* minicircle‐loaded RBCEVs for bladder cancer immunotherapy.

We further evaluated the immune functions of IL‐12 in vivo. qPCR analysis of the dissociated tumour tissues showed that the delivery of *Il‐12* minicircle‐loaded RBCEVs promoted the up‐regulation of pro‐inflammatory *Ifng*, *Il‐1b* and co‐stimulatory signal *Cd86* in the tumours (Figure 5A). Additionally, flow cytometry analysis demonstrated a substantial increase in the infiltration of total macrophages (CD11b^+^F4/80^+^), CD80^+^ macrophages (CD11b^+^F4/80^+^CD80^+^), CD4^+^ T cells (CD3ε^+^CD4^+^), CD8^+^ T cells (CD3ε^+^CD8^+^), total myeloid‐derived suppressor cells (MDSCs) (CD11b^+^Gr‐1^+^) and MHC class II^+^ MDSCs (CD11b^+^Gr‐1^+^MHC‐II^+^) in the tumours treated with *Il‐12* minicircle‐loaded RBCEVs compared to the control (Figure 5B–D). Taken together, the intratumoral administration of *Il‐12* minicircle‐loaded RBCEVs effectively inhibited bladder cancer tumour growth, enhanced immune responses and promoted immune cell infiltration (Figure 6).

## 4 DISCUSSION

IL‐12 exhibits remarkable antitumor properties and has shown great promise in preclinical research. However, its performance in clinical trials has been disappointing due to dose‐limiting toxicity and insufficient levels of IL‐12 in the tumour microenvironment.^2^, ^3^ Using local delivery to directly administer the cytokine to the tumour site could potentially address concerns regarding high toxicity and limited efficacy encountered thus far.^3^, ^8^ Multiple trials have shown decreased toxicity alongside enhanced antitumor response when restricting IL‐12 delivery to the tumour site.^3^, ^8^, ^20^ In the context of immunotherapy for bladder cancer, the bladder's natural shape as a hollow organ makes it a suitable candidate for localized cytokine delivery.^21^

We chose intratumoral delivery of RBCEVs to localize the therapeutic effect directly within the tumour site. This method inherently reduces the exposure of RBCEVs to normal tissues and minimizes systemic side effects. The localized administration ensures that the RBCEVs are concentrated in the tumour microenvironment, enhancing their interaction with cancer cells.

Additionally, cancer cells are known to have a higher rate of endocytosis compared to normal cells due to their rapid division and altered metabolism.^22^, ^23^ This increased endocytic activity facilitates the internalization of nanocarriers and EVs, enhancing the delivery of therapeutic cargo to tumour cells. Furthermore, the rapid division of cancer cells leads to increased plasmid expression. Enhanced nuclear localization and transfection efficiency in dividing cells have been observed with various carriers, including liposomes, polyethyleneimine and nanoparticles.^24^, ^25^, ^26^ This preferential uptake and processing of therapeutic agents by rapidly dividing cells increases delivery to tumour cells. Our strategy of intratumoral delivery combined with the naturally higher uptake and expression of plasmids by rapidly dividing tumour cells can help enhance the therapeutic effect on tumour cells.

There are different strategies for localized IL‐12 delivery such as immunocytokines, plasmid‐based, mRNA‐based, virus‐based and cell‐based delivery.^3^ Delivery of plasmid DNA encoding IL‐12 could offer a potentially less toxic alternative, while sustaining IL‐12 expression. Nonetheless, its application is restricted by the need for a safe and effective delivery vehicle.

We have previously shown that RBCEVs can deliver different types of RNA and antisense oligonucleotides; however, their potential for the delivery of plasmids has yet to be explored.^13^, ^14^, ^15^ In this study, we investigated the therapeutic potential of RBCEVs for *IL‐12* gene delivery in the form of minicircle DNA in bladder cancer treatment. Minicircle vectors are smaller than conventional plasmids and contain reduced bacterial sequences, allowing for low toxicity, improved uptake and higher gene expression.^27^ For the first time, we showed that RBCEV‐mediated delivery of minicircles led to transgene expression. The inclusion of the SV40 enhancer known for its nuclear targeting properties, and WPRE element involved in RNA processing and nuclear export helped enhance transfection efficiency.^28^, ^29^, ^30^, ^31^ We demonstrated that delivery of *Il‐12* minicircle using RBCEVs led to IL‐12 expression in MB49 bladder cancer cells and expression within subcutaneous tumours in mice bearing bladder cancer in the flank region.

IL‐12 is a potent pro‐inflammatory cytokine that enhances the body's immune response against tumours, primarily through the induction of IFNγ.^2^, ^3^, ^4^ IFNγ is an important cytokine that mediates the cytotoxic activity of T cells against cancer cells and promotes the recruitment of additional immune cells to the tumour site, further amplifying the immune response.^2^, ^3^, ^4^ The sustained expression of IFNγ has been shown to be correlated with clinical response.^32^, ^33^ Notably, we demonstrated that the delivery of *Il‐12* minicircle using RBCEVs stimulated IFNγ production. Additionally, we observed a significant increase in IL‐1β and CD86 levels, indicating a shift towards a pro‐inflammatory tumour microenvironment that enhances immune‐mediated killing of cancer cells.^32^, ^33^ We further showed an enrichment of CD4^+^ and CD8^+^ T cells as well as total and CD80^+^ macrophages, confirming immune cell infiltration in tumours treated with *IL‐12* minicircle‐loaded RBCEVs. Interestingly, a rise in total MDSCs was accompanied by an increase in the proportion of MHC‐II^+^ MDSCs following *Il‐12* minicircle‐loaded RBCEVs treatment, suggesting a shift in phenotype towards a more activated state and amplification of immune response in the tumour microenvironment.^34^ Importantly, intratumoral delivery of *Il‐12* minicircle‐loaded RBCEVs to a mouse subcutaneous MB49 bladder cancer model suppressed tumour growth, indicative of effective immune‐mediated tumour eradication. The observed increase in inflammatory markers and recruitment of immune cells within the tumours of mice treated with *Il‐12* minicircle‐loaded RBCEVs resulted in a robust local immune response leading to tumour reduction. This displays the efficacy of our RBCEV platform for delivery of *Il‐12* minicircle for secretion of biologically active IL‐12, stimulation of immune responses and antitumor activity.

Additionally, commonly used gene delivery vehicles such as AAVs are toxic and not redosable due to their tendency to induce host immune responses, resulting in the production of neutralizing antibodies.^3^, ^9^, ^11^ In contrast, we demonstrated that the intratumoral delivery of plasmid‐loaded RBCEVs on a repeated dosing regimen did not significantly alter mouse body weight or liver and kidney function. Therefore, our study highlights the potential of RBCEVs as a safe and redosable delivery vehicle for gene therapy.

While this study used a subcutaneous bladder cancer model, future investigations may incorporate an orthotopic model for instillation of RBCEVs into the bladder. This is important because the presence of urine and the penetration of loaded RBCEVs within the tumour microenvironment may differ between these two models.

Overall, the pleiotropic activity and robust antitumor effect of IL‐12 makes it a promising candidate for cancer immunotherapy. However, the anticipated clinical translation of IL‐12 is hindered by serious adverse effects. In this study, we demonstrate the promising potential of RBCEVs as an effective, safe and redosable nucleic acid drug delivery platform for IL‐12.

## AUTHOR CONTRIBUTIONS

Z.W., W.L. and M.T. contributed equally to this work. M.T.N.L., E.C., Q.W. and R.M. involved in study conception and funding. Z.W., W.L., M.T. and M.T.N.L. involved in study design. Z.W., W.L., M.T., F.H.Y.X. and H.S. involved in acquisition of data. Z.W., W.L., M.T. and M.T.N.L. involved in analysis and interpretation of data. Z.W., W.L., M.T., F.H.Y.X., H.S and M.T.N.L. involved in manuscript writing.

## FUNDING INFORMATION

This study is funded by the Ministry of Education (Grant NUHSRO/2021/103/RO5+6/Seed‐Sep/06), Economic Development Board (EDB‐IPP scholarship for Melissa Tan) and Carmine Therapeutics.

## CONFLICT OF INTEREST STATEMENT

MTNL is a scientific cofounder and advisor, and Melissa Tan is a research associate at Carmine Therapeutics, a company that develops gene therapy. Other authors do not have any potential conflict of interest.

## Supporting information


Table S1.

## Data Availability

The data that support the findings of this study are available from the corresponding author upon reasonable request.
