# Supplementary material for: IL‐12 minicircle delivery via extracellular vesicles as immunotherapy for bladder cancer
Source: Cell Prolif. 2024 Aug 28;58(1):e13739. doi: 10.1111/cpr.13739 (PMC11693561; doi:10.1111/cpr.13739)
Supplement: Supplementary file 1 — Table S1. [file CPR-58-e13739-s001.docx]

**Supplementary Figures**

**Table S1: List of mouse primers**

| **Genes** | **F/R** | **Sequence** |
| --- | --- | --- |
| *Gapdh* | F | AGGTCGGTGTGAACGGATTTG |
|  | R | TGTAGACCATGTAGTTGAGGTCA |
| *Il-12* | F | AGTGGGCATGTGTTCCCTG |
|  | R | GAGGCTGTTGGAACGCTGA |
| *Ifnγ* | F | GCCACGGCACAGTCATTGA |
|  | R | TGCTGATGGCCTGATTGTCTT |
| *Il-6* | F | TCTATACCACTTCACAAGTCGGA |
|  | R | TCTATACCACTTCACAAGTCGGA |
| *Il-1β* | F | GCAACTGTTCCTGAACTCAACT |
|  | R | ATCTTTTGGGGTCCGTCAACT |
| *Tnfα* | F | CCTGTAGCCCACGTCGTAG |
|  | R | GGGAGTAGACAAGGTACAACCC |
| *Cd86* | F | ACGTATTGGAAGGAGATTACAGCT |
|  | R | TCTGTCAGCGTTACTATCCCGC |
